# Dual aortic aneurysms with coronary artery and multiple cerebrovascular stenoses

**DOI:** 10.1002/ccr3.4087

**Published:** 2021-03-30

**Authors:** Masafumi Hashimoto, Kenji Mogi, Manabu Sakurai, Kengo Tani, Shuntaro Ito, Yoshiharu Takahara

**Affiliations:** ^1^ Division of Cardiovascular Surgery Funabashi Municipal Medical Center Heart and Vascular Institute Funabashi Japan

**Keywords:** abdominal aortic aneurysm, cardiopulmonary bypass, thoracic aortic aneurysm

## Abstract

Total debranching thoracic endovascular aortic repair is useful for avoiding neurological complications in cases where cardiopulmonary bypass is difficult and for devising an intraoperative cervical branch reconstruction method.

## Graphical abstract

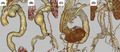

## 1 INTRODUCTION

We treated a patient with thoracic and abdominal aortic aneurysms, multiple cerebral artery stenoses, and coronary artery stenosis. Total debranching thoracic endovascular aortic repair is useful for avoiding neurological complications in cases where cardiopulmonary bypass is difficult by devising an intraoperative cervical branch reconstruction method.

Simultaneous thoracic and abdominal aneurysms account for approximately 10%–20% of all aortic aneurysms.^1^ The choice between simultaneous or staged therapy for effective treatment of multilevel aortic aneurysms remains debatable. We treated a patient with thoracic and abdominal aortic aneurysms (TAA and AAA, respectively), multiple cerebral artery stenoses, and coronary artery stenosis. Here, cardiopulmonary bypass (CPB) was difficult because of multiple stenosis of the cerebral arteries. We performed total debranching thoracic endovascular aortic repair (Td‐TEVAR), endovascular aortic repair (EVAR), and off‐pump coronary artery bypass grafting (OPCAB).

## 2 CASE REPORT

**FIGURE 1:**
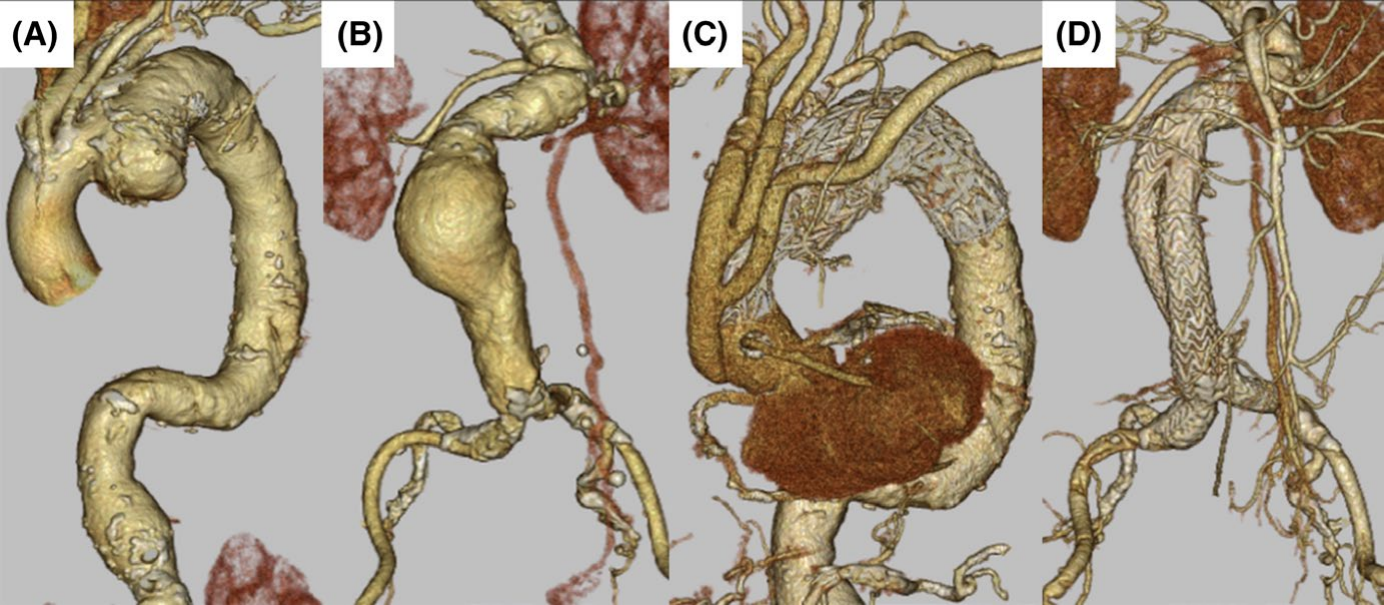
A CT angiogram reveals a saccular TAA (70 mm) (A) and an infrarenal AAA (65 mm) (B). The postoperative CT angiogram shows total debranching thoracic endovascular aortic repair of the TAA (C) and endovascular aortic repair of the AAA (D), excluding the aortic aneurysm. The patency of the great SVG is also confirmed (C). AAA, abdominal aortic aneurysm; CT, computed tomography; SVG, saphenous vein graft; TAA, thoracic aortic aneurysm

**FIGURE 2:**
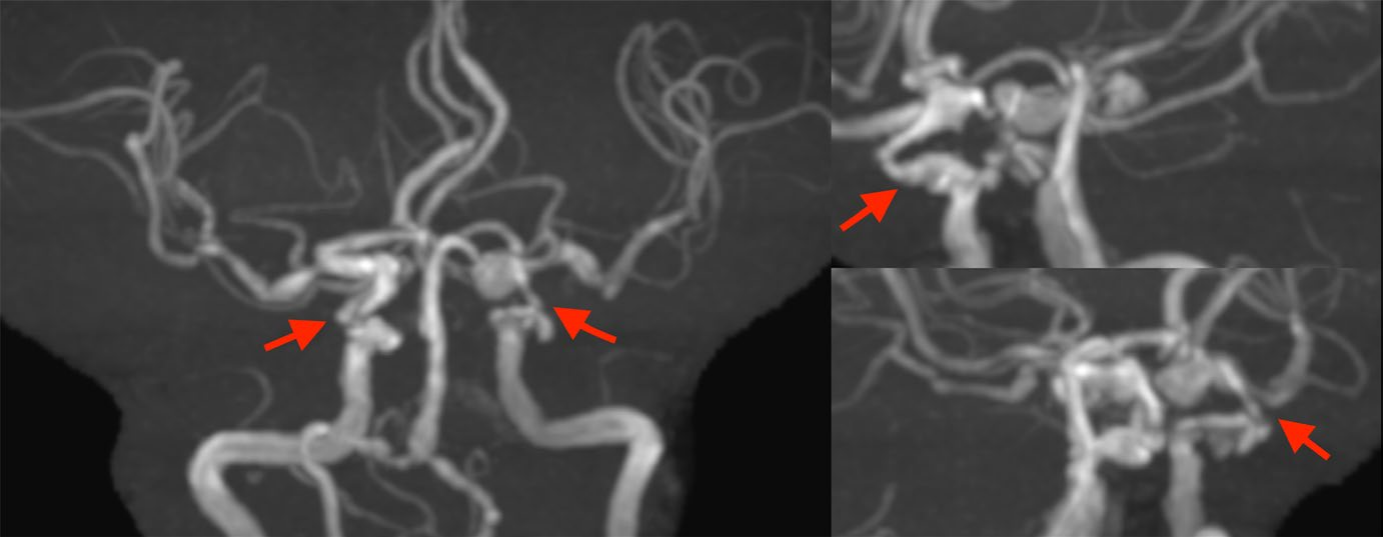
Brain magnetic resonance imaging revealed multiple stenosis of the cerebral arteries, mainly in the bilateral internal carotid artery siphon site (arrow)

**FIGURE 3:**
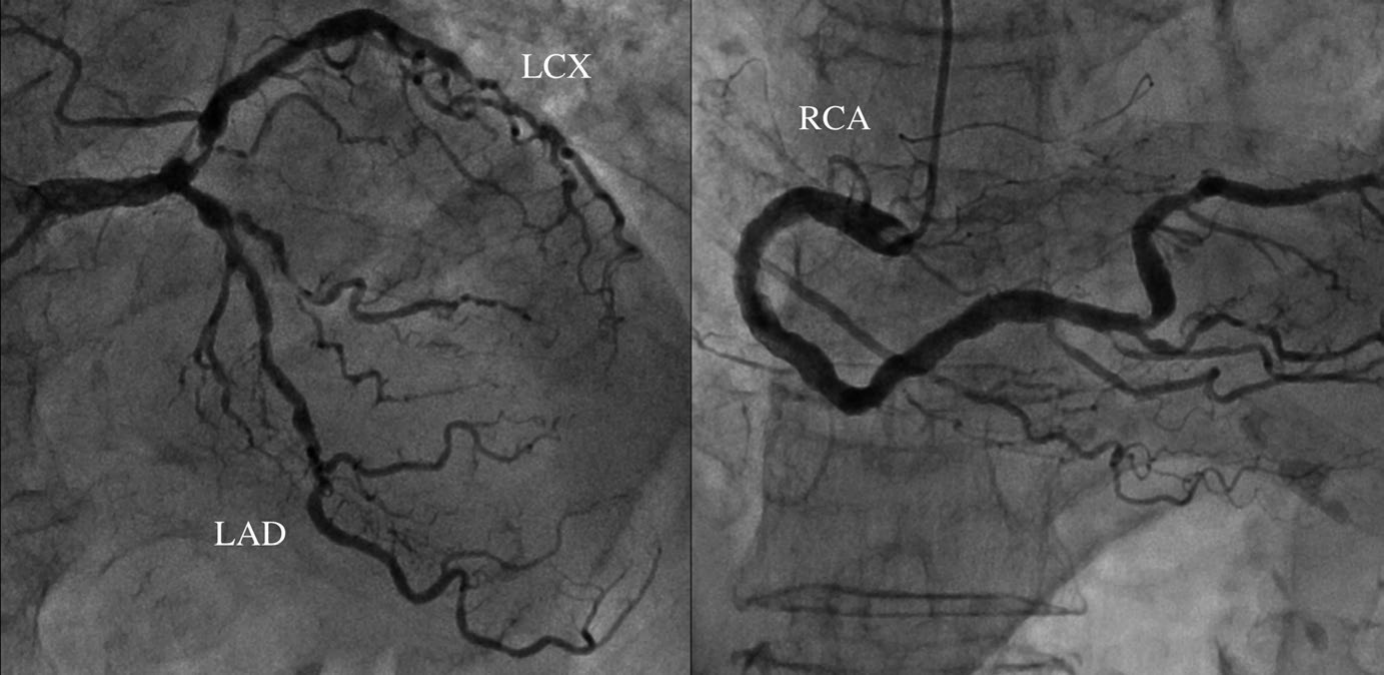
Cardiac catheterization revealed coronary artery stenosis. LAD #7, #8, and #9:75%, 75%, and 90%, respectively. LCX: 90% RCA: 4PD: 75%. LAD, left anterior descending artery; LCX, left circumflex artery; 4PD, posterior descending artery; RCA, right coronary artery

A 79‐year‐old female who presented with complaints of “pulsation” in her abdomen was referred to our hospital for suspected AAA. Her medical history comprised hypertension and dyslipidemia. On computed tomography, AAA and a saccular TAA were observed (Figure 1A,B). Immediate surgery was planned due to the high risk of rupture. However, multiple stenotic lesions were observed in the major cerebral arteries (Figure 2). Moreover, triple‐vessel disease was also observed on coronary angiogram (Figure 3). Brain magnetic resonance imaging findings suggested surgery using CPB, which carried a high risk of cerebral infarction. We planned to perform OPCAB through the left anterior descending artery (LAD) for coronary revascularization. Percutaneous coronary intervention (PCI) was planned afterward on other lesions. We planned simultaneous surgery with OPCAB from the aorta through the LAD using a great saphenous vein graft (SVG), Td‐TEVAR, and EVAR. The patient provided informed consent for the publication of this report.

### 2.1 Surgical findings

Using a median sternotomy, both axillary arteries (AxiA) were exposed. The ascending aorta was partially clamped, and end‐to‐side anastomosis was performed using a 3‐branched HEMASHIELD PLATINUM (⌀12 × 8 × 8 × 300 mm) Woven Double Velour Aortic Branch. The SVG was anastomosed using a PAS‐Port proximal automatic anastomosis system due to the limited space in the aorta, and the distal side of SVG was anastomosed to the LAD. Subsequently, an anastomosis was made to the right AxiA using another HEMASHIELD (⌀8 × 300 mm) to maintain cerebral blood flow under a beating heart. In this state, the brachiocephalic artery was blocked, and the first branch of the ⌀12 mm HEMASHIELD was anastomosed to the brachiocephalic artery. After blood flow restoration to the brachiocephalic artery, bypass to the right AxiA was blocked and cutoff. In the left common carotid artery, a catheter was inserted into the third branch of the ⌀12 mm HEMASHIELD. An end‐to‐end anastomosis was performed with the second branch of the ⌀12 mm HEMASHIELD while using the catheter to ensure blood flow to the left common carotid artery. Finally, the left subclavian artery was reconstructed by anastomosing the third branch of the ⌀12 mm HEMASHIELD into the left AxiA. After reconstruction, the left subclavian artery was ligated at the root.

Following this, TEVAR was performed from zone 0 using a Conformable GORE TAG Thoracic Endoprosthesis (WL Gore & Associates, Flagstaff, Ariz; TGU373715J + TGU373720J), and EVAR was performed using a GORE EXCLUDER AAA Endoprosthesis (W. L. Gore & Associates; RLT281418J + PLC161400J). No endoleak was observed during intraoperative imaging.

### 2.2 Postoperative course

There were no neurological complications, and the patient was extubated on the day of surgery. Although this involved simultaneous operation of Td‐TEVAR and EVAR, no deterioration of renal function was observed. The absence of endoleak and SVG patency was confirmed using postoperative computed tomography (Figure 1C,D).

## 3 DISCUSSION

Here, TAA and AAA were observed simultaneously. The rupture of untreated infrarenal aneurysms accounts for 30% of deaths after thoracic pathology repair.^2^ Furthermore, simultaneous EVAR in the thoracic and abdominal aorta was not associated with an increased incidence of spinal cord ischemia and, in fact, was associated with fewer complications and deaths than simultaneous or staged open thoracic and abdominal repairs.^3^ Thus, we performed simultaneous Td‐TEVAR and EVAR.

The standard treatment for TAA is total arch replacement, which is always performed using CPB and selective cerebral perfusion. The risk of postoperative neurological complications increases in patients with bilateral internal carotid artery stenosis using CPB.^4^ Therefore, to avoid neurological complications, we opted for TEVAR. Intraoperative cervical branch reconstruction was used to maintain cerebral blood flow under a beating heart and avoid hypoperfusion.^5^

Complete revascularization of all three branches by bypass surgery was also considered. However, heart displacement is necessary during bypass to the right coronary artery. For our patient, the left circumflex artery (LCX) and blood pressure reduction were key concerns. Intraoperative hypotension due to heart displacement can cause neurological complications. After discussion, we considered simultaneous LAD and LCX PCI; however, the stenotic lesion in the LCX was close to the LCX ostium; therefore, the stent might slip out into the left main coronary trunk, precluding a safe PCI. Therefore, we decided to use an SVG to perform bypass to the LAD and perform PCI in other areas on a different day.

The left internal thoracic artery was not used because of concerns related to coronary artery ischemia caused by coronary subclavian steal syndrome^6^ secondary to stenosis or sudden occlusion in the anastomosed part of the left AxiA.

## 4 CONCLUSIONS

In conclusion, Td‐TEVAR is useful for avoiding neurological complications in cases where CPB is difficult and for devising an intraoperative cervical branch reconstruction method. Although we encountered several challenges, the patient was treatable, without complications by developing a better method.

## CONFLICT OF INTEREST

The authors certify that there are no conflicts of interest to disclose.

## AUTHOR CONTRIBUTIONS

MH: involved in corresponding author. KM: involved in approval of the manuscript. MS: involved in approval of the manuscript. KT: drafted of the manuscript. SI: involved in data collection. YT: involved in critical revision of the manuscript.

## INSTITUTIONAL REVIEW BOARD APPROVAL OR WAIVER

The independent ethics committee of Funabashi Municipal Medical Center oversaw this project.

## PATIENT CONSENT STATEMENT

The patient provided informed consent for the publication of this report.
